# HIV-1 Latency: An Update of Molecular Mechanisms and Therapeutic Strategies

**DOI:** 10.3390/v6041715

**Published:** 2014-04-14

**Authors:** Angela Battistini, Marco Sgarbanti

**Affiliations:** Department of Infectious, Parasitic and Immune-Mediated Diseases, Istituto Superiore di Sanità, Viale Regina Elena, 299, 00161 Rome, Italy; E-Mail: marco.sgarbanti@iss.it

**Keywords:** HIV, latency, mechanisms of latency, reactivating compounds, immune therapy

## Abstract

The major obstacle towards HIV-1 eradication is the life-long persistence of the virus in reservoirs of latently infected cells. In these cells the proviral DNA is integrated in the host’s genome but it does not actively replicate, becoming invisible to the host immune system and unaffected by existing antiviral drugs. Rebound of viremia and recovery of systemic infection that follows interruption of therapy, necessitates life-long treatments with problems of compliance, toxicity, and untenable costs, especially in developing countries where the infection hits worst. Extensive research efforts have led to the proposal and preliminary testing of several anti-latency compounds, however, overall, eradication strategies have had, so far, limited clinical success while posing several risks for patients. This review will briefly summarize the more recent advances in the elucidation of mechanisms that regulates the establishment/maintenance of latency and therapeutic strategies currently under evaluation in order to eradicate HIV persistence.

## 1. Introduction

**Figure 1:**
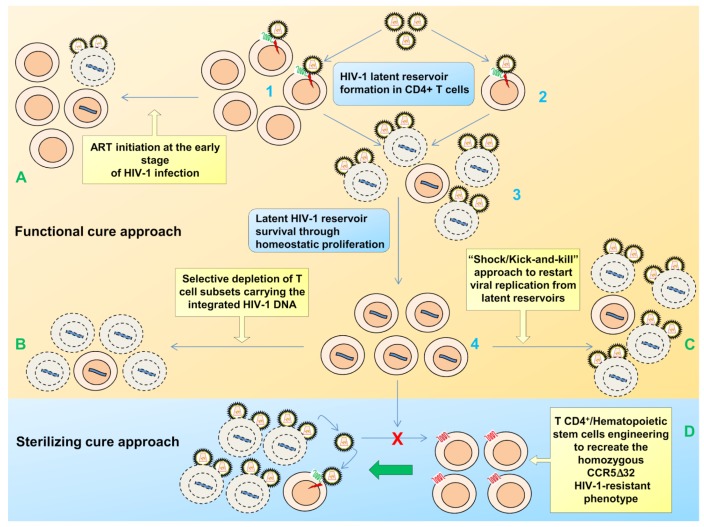
Establishment of post-integration latency and therapeutic approaches. Infection of activated CD4^+^ T cells by HIV-1 mostly results in their rapid death by the cytopathic effect of the virus. A minority of activated CD4^+^ T cells, however, becomes infected just as they are undergoing the transition from an activated to a resting-memory state where the provirus becomes silent. As an alternative, latent infection may arise from direct infection of resting CD4^+^ T cells. The established latent reservoir in the T CD4^+^ resting memory compartment then survives through homeostatic proliferation. Two major approaches to cure HIV-1 infection are the “functional cure” and the “sterilizing cure”. The “functional cure” approach includes different strategies as (I) initiation of ART during the very early primary stage of acute HIV-1 infection, leading to a long lasting control of viremia following c-ART discontinuation; (II) selective depletion of discrete T cell subsets carrying the integrated HIV-1 DNA without viral reactivation; and (III) the so called “shock/kick and kill” strategy consisting in inducing, through drugs, transcription of quiescent, replication-competent HIV-1 provirus (the “shock/kick” phase), in the presence of ART (to block viral spread), making virus reactivating cells susceptible to immune clearance, cytopathic effects and/or the effects of *ad hoc* therapeutics (the “kill” phase). The “sterilizing cure” approach, manly consists in the engineering of patient’s own T CD4^+^ cells or hematopoietic stem cells through the generation of a CCR5 deletion followed by an autologous infusion/transplant with these cells that are resistant to infection and may take over the original infected cell population.

Thirty years after the isolation of HIV-1 [1], soon after recognized as the cause of AIDS [2,3,4], and despite intensive efforts to understand the mechanisms leading to HIV pathogenesis and the remarkable achievements in treatment, the AIDS pandemic has not been defeated yet and the virus continues to spread especially in developing countries where the economic burden of life-long treatments is untenable. More than 34 million people are currently estimated to be living with HIV infection or AIDS (WHO, UNICEF, and UNAIDS. Progress Report 2012 [5]) with an overall increase over the past 10 years, and the number is expected to rise. Two third of infected individuals live in sub-Saharan Africa, South and Southeast Asia have the second highest number of people living with HIV-1, followed by the Caribbean, Eastern Europe and Central Asia where 1.0% of adults were living with HIV in 2011. Continuous research has led to the development and licensing for clinical use of more than 30 different anti-HIV-1 active compounds belonging to six different drug families that target different steps in the viral life cycle. Combination antiretroviral therapy (c-ART) has enormously reduced morbidity and mortality, transforming, *de facto*, AIDS from an incurable to a treatable chronic disease. Although antiretroviral therapy works well in the majority of patients, life-long pharmacological treatments pose severe concerns in terms of toxic side effects, development of virus resistance and huge financial load untenable for less-developed countries where the epidemic is hardest [6,7,8,9]. More importantly, despite that c-ART can routinely keep viral load under detectable levels [10,11,12], it has proven unable to cure an established HIV infection. A certain degree of chronic immune activation and inflammation is still present that, coupled with peripheral lymphoid tissues damage, represents an additional factor contributing to HIV pathogenesis [13,14,15]. As soon as therapy is interrupted, rebound of viremia and recovery of systemic infection occurs [16,17,18,19]. This is due to the existence of long-lived HIV-1 reservoirs (reviewed in [20]), established very early during primary infection and extremely stable [21]. In these reservoirs the integrated provirus is quiescent due to transcriptional silencing and not affected by c-ART that only targets actively replicating virus. Latently infected cells do not express viral proteins and hence remain invisible to the immune system. If activated, however, these cells promote new rounds of viral replication, with systemic infection (Figure 1).

Given the present failure in the development of a protective vaccine and the recognition that intensification of ART is unlikely to lead to eradication [22] novel therapeutic approaches that can eliminate persistent virus overcoming the need of lifelong adherence to antiretroviral drugs are actively pursued. Two major approaches to cure HIV-1 infection include curative strategies aimed at eliminating the latent virus reservoirs—sterilizing cure-, or at reducing the reservoirs, reaching a drug-free control of infection-functional cure-. In the latter case, in the absence of ART, a host-mediated control of the infection should restore effective immune functions lowering excessive HIV-induced immune activation and inflammation [23,24]. The first approach, a sterilizing cure, would tend to replicate the famous but unique case known as the “Berlin patient” who became undetectable for the virus after an allogenic bone marrow transplantation from an homozygous CCR5∆32 donor [25,26,27]. A functional cure is instead spontaneously achieved in a rare population of individuals known as “Elite Controller” (ECs) and has been, recently, reproduced in the VISCONTI cohort, where 14 HIV patients following discontinuation of c-ART maintained long lasting control of viremia [28]. These patients called post-treatment controllers (PTCs) are distinct from ECs in that they lack the protective HLA B alleles and accordingly, had poorer CD8^+^ T cell responses and more severe primary infections than the ECs had. At variance with most patients that begin ART treatment in the chronic phase of infection, however, PTCs initiated ART during the very early primary stage of acute HIV infection. Other studies have also demonstrated that ART is more efficient at reducing the size of residual reservoirs when initiated early after HIV infection [29].

Current approaches to a functional cure thus include: (i) ART initiation at the early stage of HIV-1 infection [30,31,32,33,34]; (ii) specific depletion of discrete T cell subsets carrying the integrated HIV-1 DNA without viral reactivation [35,36,37]; (iii) elimination of the latent reservoir through the so-called “shock-and-kill” or “kick and kill” approach. This approach consists in inducing transcription of quiescent, replication-competent HIV provirus (the “shock/kick” phase), in the presence of ART (to block viral spread), making virus-reactivating cells susceptible to immune clearance, cytopathic effects and/or *ad hoc* therapeutics (the “kill” phase) [38,39,40] (Figure 1).

This review will briefly overview our current understanding of HIV-1 latency/persistence despite ART, and recent advances in translational approaches used or proposed to escape from latency.

## 2. Viral Persistence and Reservoirs

After acute infection, HIV-1 becomes latent in a fraction of infected cells, while it continues to replicate in others.

The presence of latent HIV-1 infection reservoirs was suggested initially when ongoing viremia at levels up to 50 copies per milliliter was still observed in patients on c-ART, despite prolonged suppression of HIV replication. In addition, a number of patients experienced transient episodes of viremia, or HIV-1 “blips”, even with suppression of the viral load for many years [41,42,43,44]. The source of this persistent viremia is still debated and may be the result of multiple mechanisms. These include low-level of ongoing viral replication due to incomplete inhibition of viral replication by c-ART [45,46] the existence of viral sanctuary poorly accessible to drugs as cells of the nervous system [47], the genital tract, the gut [48], other immune cells including the monocyte/macrophage lineage [20,49,50] and the more recently identified and still debated hematopoietic cell compartment [51,52,53]. In these cell types or anatomical sites, it is however, still debated whether or not viral persistence is due to true latency or to low level ongoing replication [54,55]. In particular, cells of the monocyte/macrophage lineage together with CD4^+^ T cells are the primary targets for HIV-1 infection *in vivo*, and HIV-1-infected monocytes have been identified in the peripheral blood of viremic and HAART-treated patients [56]. Evidence of sequence evolution in comparison to resting CD4^+^ T cells, indicates their distinct contribution to plasma viremia [57,58]. The *in vivo* reactivation of these infected macrophages in response to opportunist infections [59] would also be in favor of macrophages as HIV-1 reservoirs. These cells however, behave differently from T cells in that macrophages are more resistant as compared to T lymphocytes to viral cytopathic effects and maintain low levels of viral replication [20,60]. Antiretroviral therapy also works differently in the two cell types. Recently, it has been reported that resistance to the HIV integrase inhibitor raltegravir follows a single-step pathway (a single mutation) in macrophages compared to T cells where multiple mutations are required to obtain resistance [61]. Thus, macrophages could function as incubators of virus resistant strains that can be transferred to CD4^+^ T cells after their recruitment in different tissues including the gut and other, so-called sanctuaries, such as the brain.

Resident macrophage/microglial cells are, indeed, the main targets for HIV-1 in the CNS. Due to the blood-brain barrier that prevents an easy access to antiretroviral drugs, the brain is considered an ideal reservoir for HIV-1. In the brain the virus adapts and infects macrophage/microglia and also astrocytes, all long-lived cells, where it causes minimal cytopathology [62]. Recently, it has been reported that the HIV-1 LTR repression in astrocytes is subject to at least some mechanisms reported to induce LTR repression in T cells, including the activity of HDACs and HMTs [63]. However, whether these cells fit the stringent definition of latent infection with the capacity of the integrated genomes to be reactivated *in vivo* to produce infectious virus capable to reinitiate the disease [64] is still unknown. Nevertheless, the so far proposed eradication therapies for peripheral lymphoid reservoirs illustrated in the following sections, have, however, to take into account that they may result in highly deleterious consequences if a viral reservoir has been established in the brain. Thus a tailored approach should be considered that avoids reactivation in this compartment with potential consequent episodes of encephalitis and brain damage.

In support of the on-going viral replication hypothesis, there are ART intensification studies using the integrase inhibitor raltegravir that showed the accumulation of 2-LTR (long terminal repeats) circles in the PBMCs of a certain percentage of treated patients [65].

A major source of persistent viremia is, however, essentially represented by the episodic reactivation of the virus from the long-lived memory CD4^+^ T cells. In favor of this hypothesis are phylogenetic studies revealing how the residual circulating virus is genetically stable [66,67], even if, as outlined above, other studies found HIV-1 sequences that were not present in the resting CD4^+^ T cell population [68,69].

Stably integrated but latent HIV-1 genomes were found more than 15 years ago in resting memory CD4^+^ T cells *in vivo* [44,70,71,72,73]. Currently, these cells are thought to be the major reservoir of post-integration latent virus and as such they are currently the major focus of investigations.

Since a fully resting T cell is not permissive in the early steps of viral replication, a current model of latency establishment postulates that the latent infection may arise from infrequent infection of an antigen (Ag)-activated memory CD4^+^ T cell, even if direct infection of resting CD4^+^ T cells has also been proposed [74]. Infection of activated CD4^+^ T cells mostly results in the rapid death of activated T cells by the cytopathic effect of the virus, but rarely, or a minority of them, become infected just as they are undergoing the transition from activated to resting-memory state where the provirus becomes silent, reviewed in [75] (Figure 1).

Although small in number (approximately one in 10^6^ resting CD4^+^ T cells containing fully integrated and transcriptionally silent HIV proviruses predicting that a patient may harbor a number of <10^7^ latently-infected T cells), these latently infected cells possess an extremely long half-life that mostly contributes to the life-long persistence of HIV even in patients on c-ART. By direct longitudinal analysis of the decay rate of this latent reservoir, the half-life of latent cells producing replication-competent virus was estimated in 44 months. In the absence of any *de novo* infection and of strategies to eliminate latent virus, natural eradication would thus take more than 60 years of uninterrupted treatment [21,76].

Among different T CD4^+^ subsets both long-lived central memory (TCM) and transitional memory (TTM) cells represent the majority of the reservoir with relative contribution of the two subsets varying from one patient to another [77]. A viral reservoir of limited size mainly consisting of TCM cells is present in patients who began an early treatment and have normal T CD4 counts, while patient with low T CD4 counts have a reservoir consisting mainly of TTM cells. These reservoirs that are established within the first week from the beginning of the infection are maintained by different mechanisms: through T cell survival and low-level antigen-driven proliferation (TCM) and by IL-7-mediated homeostatic proliferation (TTM) and hence the reservoir does not decay with time [77,78]. Intensification of therapy with integrase inhibitor, while resulting in a transient increase in episomal DNA in some ART-suppressed patients [65], does not appear to reduce the reservoir size [22,79]. Moreover, recent data suggest that the actual number of latently infected cells bearing a replication-competent virus, could be almost 60-fold larger than previously estimated [80]. Indeed, by characterizing proviruses present in viral outgrowth assays not induced by maximal PHA stimulation [81], the Siliciano lab showed that around 12% of the non-induced proviruses that were generally considered defective, instead, displayed intact genomes, normal long terminal repeat functions and were replication-competent. This seems to indicate a stochastic driver of HIV-1 escape from latency [80], further challenging the hope of reservoir elimination only by the currently suggested “shock and kill” strategy.

## 3. Mechanisms of Post Integration HIV-1 Latency

**Figure 2:**
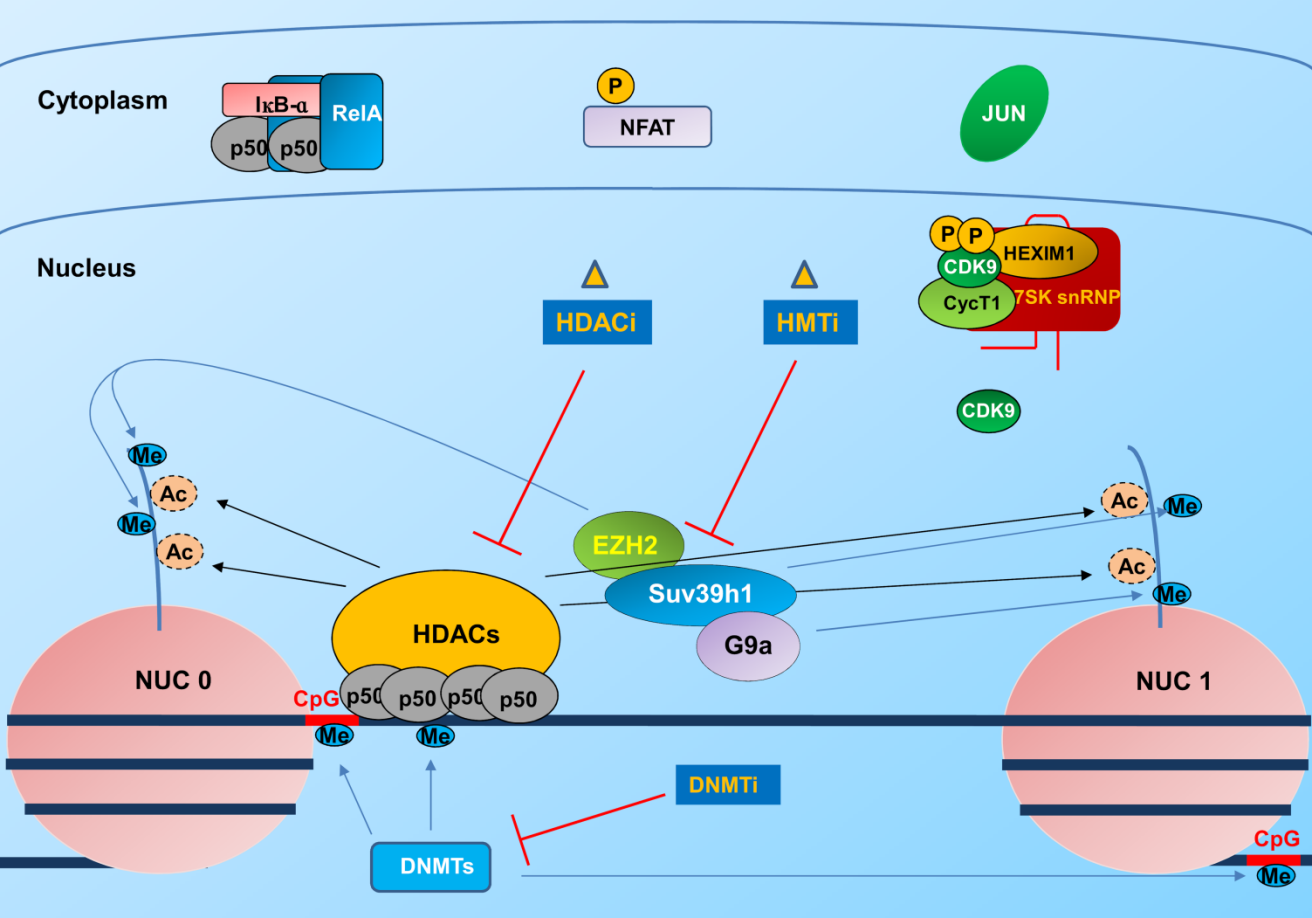
HIV-1 gene transcription is silenced in latently infected cells: epigenetic mechanisms of silencing affected by epigenetic modulators. Transcription initiation at the HIV-1 LTR is inhibited in latently infected CD4^+^ T cells due to different epigenetic silencing mechanisms. These include: recruitment of histone deacetylases (HDACs) by the NF-κB p50/p50 homodimer resulting in deacetylation of histones at the Nuc0 and Nuc1 nucleosomes, recruitment of histone methyltransferases (HMTs) as Suv39h1, EZH2 and G9a, resulting in methylation of histones and DNA methyltransferases responsible for DNA methylation at CpG islands. Crucial transcription factors responsible for initiating transcription at the LTR, such as NF-κB, NFAT and cJun (a sub unit of AP1) are then sequestered in the cytoplasm in an inactive state, contributing in the establishment/maintenance of latency. The P-TEFb factor, crucial for HIV-1 transcriptional elongation, is part of an inactive complex and together with the low amounts of the P-TEFb subunit cyclin T1 in latently infected CD4^+^ T cells, represent a further mechanism of transcriptional restriction. HDAC, HMT and DNMT inhibitors are all being explored to promote escape from latency in the context of the “shock/kick and kill” strategy.

Latency is a multifactorial process that involves multiple mechanisms and factors. Both naive and memory subpopulations of resting lymphocytes represent an extremely restrictive environment for HIV-1 replication, and although the mechanisms that lead to HIV-1 latency in CD4^+^ T cells are still not completely understood, a number of different cellular and molecular mechanisms, that contribute to the establishment and maintenance of the latent state in these cells, have been described, as also recently reviewed [82]. These mechanisms exploit both cellular and viral factors and mostly act at the level of suppression of transcription of the viral promoter LTR (Figure 2). The site of viral integration, the chromatin organization at the viral promoter, transcriptional interference, the pool of available cellular cofactors required for HIV expression and suboptimal amounts of the viral trans activator Tat, the presence of microRNA and mechanisms of RNA interference are all major factors that contribute to the transcriptional silencing of integrated HIV-1 proviruses.

### 3.1. Integration and Chromatin Organization at the Viral Promoter

Integration of HIV-1 proviral DNA into the host genome is a critical step in the viral life cycle. Curiously even if latency results in minimal or null proviral gene expression, HIV provirus has been found integrated predominantly within actively-transcribed genes, both in resting CD4^+^ T cells from HIV-infected patients on c-ART [83,84], and in a primary *in vitro* model of HIV latency [85]. This likely results from the interactions of the viral pre-integration complex (PIC) with host factors that are present at regions of active host gene expression and suggests that transcriptional interference may significantly contribute to the establishment and maintenance of latency [86]. This interference may depend on the orientation of the provirus relative to the host gene: if in the same orientation, promoter occlusion can occur with displacement of constitutively expressed transcription factors such as Sp1 binding the viral promoter [86,87]; if in opposite polarity, collision of RNA pol II complexes from the host and viral promoters determines premature termination of transcription [88].

Despite the preferential integration within euchromatin, which favors transcription, the chromatin environment and changes in the chromatin structure such as modifications of nucleosomes and DNA by acetylation and methylation may influence the establishment and maintenance of proviral quiescence by interfering with expression of viral genes [89,90,91,92,93,94,95].

Early reports indicated that, regardless of the site of proviral integration, in the transcriptional silent state, two nucleosomes, Nuc0 and Nuc1, positioned upstream of the modulatory region and downstream of the core promoter and cis-regulatory elements, respectively, assemble within the 5' long terminal repeat (LTR) of HIV-1 (Figure 2). These nucleosomes overlap with binding sites for several transcription factors key in HIV-1 gene expression [96]. Consistently, epigenetic modifications and disruption of Nuc1 are required for LTR driven transcription activation and viral gene expression both in productive infection and in models of latent infection [92,94,96]. The Nuc1 indeed, presents lysine 9 tri-methylated histone 3 (H3K9me3), heterochromatin protein 1 (HP1) and low histone acetylation, all markers of silent heterochromatin [89,92]. During latency, histone deacetylases (HDAC), specifically the class I HDACs 1, 2, and 3 [97] and histone methyltransferases (HMTs) are recruited to the viral promoter through multiple host factors (Figure 2).

These cellular factors include YY1 and LSF [98,99,100], COUP-TF interacting protein (CTIP2) [101], CBF-1 [102], NF-κB homodimers p50/p50 [103] c-myc and Sp1 [104] that recruit HDAC1. Ras-responsive binding factor 2 (RBF-2) in complex with the transcription factors TFII-I has been, instead, reported to be involved in recruiting HDAC3 to the HIV-1 LTR [105]. Also some members of the Interferon Regulatory Factor (IRF) family [106] as IRF-8 may contribute to silencing the HIV promoter via recruitment of HDAC3 or by competing with LTR activators as IRF-1 [107,108,109,110]. Consistently, IRF-8 expression is strongly down-modulated when latently infected cells are induced to viral reactivation [110].

Histone methylation may also reinforce HIV-1 latency. The HMTs EZH2, G9a and SUV39H1 have been recently reported to regulate HIV-1 transcription by inducing histone H3 methylation at lysine 9 (H3K9) and lysine 27 (H3K27) [111,112,113].

DNA methylation is also associated with gene silencing and promoter regions of HIV-1 DNA can also be methylated on cytosine residues (CpG islands). Two CpG islands flanking the HIV-1 transcription start site are present and hypermethylated in latently infected cells. Nevertheless, the role of DNA methylation in HIV-1 latency remains more controversial. In the latent reservoir of HIV-1-infected individuals without detectable plasma viremia, HIV-1 promoters and enhancers have been shown to be hypermethylated and resistant to reactivation, as opposed to the hypomethylated 5' LTR in viremic patients [93]. In support of a role of cytosine methylation is also the observation that one of the CpG islands in the HIV-1 promoter is bound by the transcriptional repressor methyl-CpG binding domain protein 2 (MDB2) and by HDAC2 [114]. However, the importance of these modifications in defining latency in patients has been recently questioned. Analysis of integrated HIV-1 genomes in resting CD4^+^ T cells from some aviremic patients indeed shows infrequent methylation, and inhibition of DNA methylation seems not required for reactivation of expression [115]. Since it is difficult to isolate the latent pool *in vivo*, assessing the exact role of these modifications in patients remains challenging.

### 3.2. Transcription and Elongation Factors Relevant for HIV-1 Gene Expression

**Figure 3:**
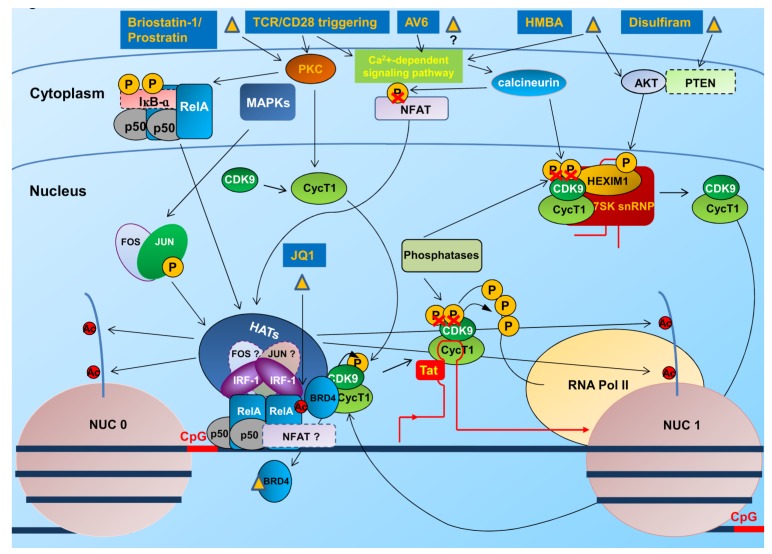
Transcriptional reactivation of HIV-1 LTR by stimulation/activation of initiation and elongation factors. Several compounds including T cell activators and differentiating agents have been identified that induce transcription of latent HIV-1 integrated genome. These act as inducers of protein PKC and NF-κB/NF-AT pathways and P-TEFb stimulators. Briostatin-1 and Prostratin are activators of PKC that once activated, phosphorylates the NF-κB-inhibitor IκB-α with its subsequent degradation and accumulation in the nucleus of the active NF-κB (RelA/p50) heterodimer. Activated PKC also triggers the MAP kinase pathway activating the AP1 dimeric factor (Fos/Jun) also recruited to the LTR enhancer upon binding to NF-κB. AV6 requires activation of NFAT to stimulate reactivation. HMBA activates the calcium pathway and calcineurin, contributing to NFAT activation, and stimulates the AKT kinase, which in turn phosphorylates HEXIM1, with the release of the P-TEFb (CycT1 and CDK-9) elongation factor from the inhibitory complex (HEXIM1/7SKsnRNP). Disulfiram stimulates the degradation of PTEN, similarly determining the activation of AKT and the release of HEXIM1. JQ1, by targeting BRD4, may release P-TEFb, promoting elongation.

HIV-1 gene expression is strongly dependent on host cell transcription factors. The 5' LTR contains multiple sites for the binding of cellular transcription factors including NF-κB, nuclear factor of activated T cells (NFAT), Sp1 and the activator protein 1 (AP1), whose activation by external stimuli starts HIV-1 transcription (Figure 3).

In addition to epigenetic restriction that impedes the binding of factors crucial for initiation and/or elongation of transcription, a limited availability of these factors may also cause a block in viral replication. The importance of transcription factor restriction for HIV latency establishment was suggested early on, when it was hypothesized that in primary T cells, HIV-1 latency is generated by viral shutdown during the transition of activated T cells to a memory phenotype [116]. In resting cells and during latency, many cellular factors are sequestered in the cytoplasm, synthesized/activated at suboptimal doses or become part of inactive protein multicomplexes (Figure 3). This is indeed the case of NF-κB family members that are present in the cytoplasm by the IκB inhibitory proteins. In the nucleus of HIV-1-infected resting cells, the inhibitory p50/p50 homodimer that lacks the transactivation domain present in the classical p50/RelA heterodimer NF-κB complex, is bound to the 5' LTR and represses transcription upon recruitment of HDAC1 thus promoting latency [103] (Figure 2). Upon T cell activation, instead the p50/RelA NF-κB heterodimer is activated following the phosphorylation of IκB-α that results in its subsequent ubiquitination and degradation by the proteasome allowing NF-κB to accumulate in the nucleus and displace the p50/p50 homodimer (Figure 3). The heterodimer p50/RelA then stimulates LTR transcription through the recruitment of HATs that remodel Nuc1 [117]. NFAT also binds LTR at the κB sites [118] and at two sets of NFAT-binding sites [119]. As NF-κB, the NFAT family members are retained in the cytoplasm of unstimulated cells and upon Ag-mediated T cell activation, translocate to the nucleus in a calcium-dependent fashion [120] recruiting HATs [121] to stimulate LTR transcription [122]. Which of these factors is more important for reactivation of latent HIV-1 proviruses is still debated and the cellular context seems to be crucial [123]. Other cellular transcription factors as AP1 and IRF-1 play a role in LTR transcription by binding to the LTR enhancer in association with activated NF-κB [109,124]. Restriction in availability of the potent viral activator of LTR transcription Tat as a consequence of small changes in initiation rates or of the presence of Tat variants with impaired transactivation activity [125], are other hallmarks of repression of HIV-1 transcription that forces the virus into entering the latent state.

Reversible acetylation of nonhistone substrates also regulates integrated latent HIV-1 proviruses. An example is the NF-κB RelA subunit that is targeted by p300/CBP for acetylation at multiple sites, including lysines 218, 221, and 310. These modifications affect overall transcriptional activity of NF-κB by regulating distinct NF-κB functions including DNA binding and assembly with IκB-α [126,127].

At the level of transcription elongation, stimulation of LTR requires the positive transcription elongation factor b (P-TEFb) complex that comprises cyclin T1 and cyclin-dependent kinase 9 (CDK9) [128,129,130]. CDK9 promotes serine 2 phosphorylation of the C-terminal domain of the largest subunit of RNA polymerase II, an event that coupled with phosphorylation of the C-terminal domain at serine 5 by the TFIIH kinase and phosphorylation of negative transcription elongation factor (N-TEF) determines the transition from the initiation to the elongation stage of transcription [131,132]. P-TEFb is sequestered in large catalytically repressed, ribonucleoprotein complex (RNP) comprising 7SK RNA and 7SK binding proteins as EXIM-1 or HEXIM-2, 7SK methylphosphate capping enzyme (MePCE) and La ribonucleoprotein domain family, member-7 (LARP7) [133,134,135,136]. Recently, and in contrast to what previously observed in dividing cells, it has been reported that, in resting CD4^+^ T cells, naïve or memory, and independent of their infection status, Cyclin T1 and T-loop-phosphorylated CDK9, are expressed at low levels and increase upon activation [137]. Thus, beside activation, P-TEFb availability may be determined by the differential expression of its subunits. Nevertheless, whatever is the mechanism of P-TEFb unavailability, its activity has to be recovered and the factor has to be recruited to LTR to overcome paused RNA polymerase II complex associated with the latent LTR promoter (Figure 3).

In productively infected cells, the viral transactivator Tat, following the binding to the transactivation-responsive element (TAR) at the 5' end of HIV transcripts, efficiently recruits active P-TEFb to the HIV-1 promoter region [128,138]. In the absence of Tat, recruitment of P-TEFb by the Raf-mediated ser276 phosphorylated RelA subunit of NF-κB has been so far described only in dendritic cells following gp120 binding to DC-SIGN [139], while the mechanism of recruitment in productively or latently HIV-infected and resting T cells remains to be defined.

In this respect, indirect evidences suggest that the cellular transcription factor IRF-1 could fulfil this task. Indeed, IRF-1 has been shown to physically interact with different HATs like CBP, p300 and PCAF [140], and with RelA contributing to the PCAF-mediated acetylation of RelA at Lys 310 in TCR stimulated CD4^+^ primary T cells [141]. In this respect, RelA acetylation is essential for the activation of a set of inflammatory genes by transcriptional elongation [142]. Moreover, IRF-1 is required for NF-κB transcriptional activity at the HIV LTR in the absence of Tat [109]. In a similar fashion, ERK-mediated activation of the AP1 transcription factor in latently infected U1 cells drives a synergistic activation of HIV-1 LTR transcription together with NF-κB, through a physical and functional interaction between the two transcription factors at the viral promoter [124]. Due to the importance, recently reported, of the AP1 binding site on the LTR in the establishment of latency [143] and the identification of the chemical JNK/AP1 pathway inhibitor AS601245 as a promoter of HIV latency, preventing the release of P-TEFb from its inactive complex with HEXIM-1 [144], it is tempting to speculate that AP1 interaction with NF-κB could play a role in the recruitment of P-TEFb.

Post-transcriptional blocks have also been involved in latency. These include impaired HIV-1 mRNA nuclear export [145] and expression of host or viral microRNAs that may impede HIV-1 mRNA expression or translation [146,147]. The contribution of miRNA to the maintenance of latency has been shown both *in vitro* and *ex vivo* [148,149] while their role in the establishment of latency is not yet fully defined [146,147].

The TAR element is itself a source of miRNAs upon recognition and cleavage by the DICER protein. These miRNAs silence gene expression by facilitating the recruitment of HDAC1 at the viral LTR [150]. Recently, it has been shown that the microprocessor complex consisting of at least two subunits, the RNase III Drosha and the dsRNA-binding protein Dgcr8, orchestrates the recruitment of termination factors such as Setx and Xrn2, and the 3'-5' exoribonuclease, Rrp6, to initiate RNAPII pausing and premature termination at the HIV-1 promoter through cleavage of TAR. Rrp6 further processes the cleavage product, generating miRNAs that mediate chromatin remodeling and transcriptional repression [151].

## 4. Therapeutic Approaches to Overcome Latency

Overall establishment and maintenance of latency is a dynamic process regulated by accumulation of different events and, once established, the expression of the proviral promoter is tightly restricted at several levels, all of which need to be overcome to restart viral production as part of the “shock and kill strategy”.

In pharmacological interventions currently under investigation, strategies that target transcription initiation and elongation factors, large screenings that identify novel compounds irrespective of their molecular target and immune-based strategies that in combination with reactivating compounds should enhance the clearance of the reactivated cells and/or restore immune functions, have all been considered.

### 4.1. Interventions to Revert the Latent State

Strategies aimed at reverting the latent state include the use of chromatin remodeling agents such as acetylation and methylation inhibitors, T cell activators and differentiating agents (Figure 2 and Figure 3).

Due to their testing in clinical trials as anticancer drugs [152] and as such extensively investigated also in terms of pharmacological and toxicological properties [153], HDAC inhibitors (HDACis) are the most advanced in the clinical testing as HIV-1 antilatency agents [154]. The first HDACi tested in patients under ART was valproic acid (VPA) a weak non-selective HDAC inhibitor approved to treat epilepsy. Initial promising results obtained with VPA indicating a significant decline in the level of latently infected CD4^+^ T cells in three out of four patients [155] were, however, not confirmed in subsequent larger studies where VPA failed to decrease the latent pool of resting CD4^+^ T cells [156]. The suberoylanilide hydroxamic acid, SAHA (vorinostat) an HDACi approved to treat cutaneous leukemia, which is active at nanomolar doses and selective for Class I HDACs, has instead shown potent activity in activating latently infected cells. SAHA was initially shown to induce simultaneously acetylation of histone H3 and transcription of HIV RNA in resting CD4^+^ T cells *in vitro* and *ex vivo* in resting CD4^+^ T cells from HIV-infected patients on suppressive c-ART [157,158]. These effects have been, recently, confirmed in a pilot single-dose trial in few aviremic patients on ART. Results have shown that this drug can disrupt latent infection within a detectable proportion of the reservoir of latent resting CD4^+^ T cells [154]. However, while these results constitute a proof-of concept that clinically exposure to an HDACi might disrupt latent HIV-1 infection, due to the criteria used to select patients in the study, the wide effectiveness of the treatment at clearing the viral reservoir is still unknown. Multiple-dose clinical studies of SAHA are currently underway that shortly should answer this concern.

It has also to be reminded that SAHA is a mutagen *in vitro* in standard bacterial assays, and as such its use in humans has been approved only for short-term exposure [159].

Novel and selective HDACis are also under investigation in order to reduce toxicity and increase specificity. These include givinostat (ITF2357) and belinostat (PXD101), that are in clinical trial as anti-inflammatory agents for juvenile iodopathic arthritis and for the treatment for various cancers [160], Panobinostat (LBH-589) that is a pan-HDACi more active than givinostat and vorinostat in reactivating latent HIV in cell culture but much more toxic [161]. Other class-selective HDACis newly synthesized or FDA-approved for different cancers that are under evaluation as HIV-1 reactivating agents include the class I selective HDAC1 Oxamflatin [162], NCH-51 [163] and Romidepsin (FK228) [164]. Droxinostat instead specifically targets HDAC3 and its activity in reversing latency underlines the important role of HDAC3 in HIV-1 latency [165].

Interestingly, the action of HDACis has been shown to be not limited to histone acetylation. HDACs can, indeed, deacetylate an increasing number of nonhistone proteins that impact diverse cellular processes. Consistently, HDACs target a variety of transcription factors as well as other nuclear and cytoplasmic proteins [166]. These include the RelA subunit of NF-κB that is acetylated at lys 221 and 218 by p300, upon NF-κB activating stimuli promoting NF-κB activity [127]. This may explain the synergism in reactivating latent HIV-1 observed between HDACi and other reactivating agents as compounds that stimulates NF-κB [167]. In spite of several advantages in the use of HDACis for HIV-1-purging strategies, including absence of proliferation and activation of T cells [168], activity in a broad range of cells, repression of CXCR4 chemokine receptor expression and function [169] and extensive testing in clinical trials as anticancer drugs [153], HDACi exact mechanism of action requires further investigations, and exact doses and administration schedules, to achieve HIV-1 induction while reducing toxicity, remain to be defined. Moreover, since HDACs are not the sole targets of these compounds, a major concern is that the generalized increase in histone acetylation they induce can determine the expression of a large number of genes whose effects are unknown. A careful and extensive testing of more selective and potentially safer HDACis, even in animal models, must thus be performed before their extensive use in humans to purge HIV-1 from latently infected cells [170,171].

Specific histone methyltransferase inhibitors as BIX01294 and chaetocin inhibitors of G9a and SUV39H1, respectively, have been reported to reactivate latent HIV-1 even if at different extent, with minimal toxicity and without causing T cell activation [112,172]. The effect of these compounds was enhanced in combination with either the HDACi SAHA or the non-tumor-promoting NF-κB inducer prostratin [112,173,174].

Another therapeutic approach considered to overcome latency consists in the recovery of levels and/or activation of transcription factors that are down regulated in latently infected cells. Many agents being considered for purging the latent reservoir act as inducers of protein kinase C (PKC) and NF-κB/NF-AT pathways and P-TEFb stimulators. As mentioned above, these factors are sequestered into inactive complexes or expressed at suboptimal levels in latently-infected cells. However, a major concern and obstacle to the use of some compounds that recover the activity of these cellular factors is the broad cell activation and off-targets effects that they may induce.

Both prostratin and bryostatin-1 are non-carcinogenic PKC activators, that have been enrolled as potentially safe anti-HIV-1 therapeutics [175]. Prostratin activates HIV transcription in several J-lat cell-line models of HIV latency and in combination with VPA and SAHA synergistically increases the amount of virus produced [167]. Although, recent practical synthesis in high quantities and a low cost of prostratin made this drug available for clinical trials [176], it is unclear whether this drug will proceed into clinical studies because of pre-clinical safety and toxicity testing.

Bryostatin-1, a macrocyclic lactone, evaluated in several clinical trials as antineoplastic agent, structurally unrelated to prostratin, has been similarly shown to reactivate latent HIV-1 via activation of both PKC and 5' adenosine monophosphate-activated kinase (AMPK) pathways alone or in combination with HDACis and importantly is non-toxic *in vitro* and does not induce T-cell activation [177,178]. Interestingly, recent data indicate that bryostatin has an indirect role also in stimulating elongation. In resting primary CD4^+^ T cells where levels of P-TEFb are very low, bryostatin-1, indeed, increased levels of this elongation factor thus reinforcing the effects of other activators as HDACis [179].

In addition to stimulate expression of latent HIV-1 by modulating the PKC pathways, prostratin and bryostatin-1 have demonstrated immune modulating capacities that may be beneficial for their use as antilatency drugs. They down regulate both the CD4 receptor and the CXCR4 and CCR5 coreceptors, thus preventing *de novo* HIV-1 infection in susceptible cells [180,181,182], but also inhibit HIV-1 *de novo* infection in a receptor dependent and independent fashion in monocytic and lymphocytic infection models [177]. Moreover, bryostatin-1 acts as a Toll-like receptor 4 (TLR4) ligand and an inducer of the macrophage inflammatory protein 1α (MIP1-α) and the RANTES chemokines [183] both known as HIV-1 suppressive chemokines against CCR5-tropic viruses [184]. Despite similarity in the activities of these two compounds that are in favour of their utilization in HIV eradication efforts, only bryostatin-1 seems ready for the clinic. In this respect, so far, clinical use of bryostatin-1 has been hampered by its, and clinically relevant derivatives, limited supply. Very recently, the synthesis of a new family of designed bryostatin analogues that effectively induce latent HIV activation *in vitro* with potencies similar to or better than bryostatin-1 has been reported, pushing testing in human clinical trials [185]. However, some side effects recently reported in a phase II clinical trial for the treatment of ovarian cancer with bryostatin-1 in combination with antineoplastic drugs [186] still require overall caution in the use of these therapeutics and for further investigations specifically in the setting of HIV-1 infection.

Recruitment of the active elongation factor P-TEFb to the viral promoter is another limiting step in viral reactivation. Among compounds that act as P-TEFb activators are Hexamethylene bisacetammide (HMBA) and the recently reported inhibitors of BRD4.

HMBA, was originally developed as an anti-cancer drug due to its pro-differentiation capacity and has been considered for anti-latency approaches as a modulator of the HMBA-inducible-1 (HEXIM1) component of the 7K small nuclear ribonucleoprotein complex. HMBA-activated PI3K/Akt pathway phosphorylates HEXIM1 releasing P-TEFb followed by its recruitment to the viral promoter in a Sp1-dependent manner even in the absence of HIV-1 Tat [187]. Interestingly, HMBA also increases the nuclear level of CDK9 and the pool of P-TEFb that is not sequestered by HEXIM1 [188]. Considering that shortage of these proteins favors latency [137], this should constitute an additional advantage. HMBA also down regulates surface expression of CD4 in PBMC and inhibits T cell activation, preventing *de novo* HIV-1 infection. In spite of these advantages, the efficacy of HMBA in reversing latency was however, shown to be very low in the primary latency cell model developed in the Siliciano lab [189] and in CD4^+^ T cells, from aviremic HIV-1 infected donors [188]. In the clinic HMBA reached phase II clinical trials for acute myelogenous leukemia [190] and in this setting dose-related thrombocytopenia was observed. Moreover, HMBA is also rapidly metabolized, eventually requiring continuous infusion to achieve detectable drug levels. Thus, despite the apparent advantages in the use of this compound to purge the latent reservoir, clinical trials with HMBA in HIV-1 patients have not been pursued to date.

A new approach, recently considered to recruit P-TEFb to HIV LTR, is inhibition of BRD4. Intriguingly, BRD4 is generally associated with gene activation through recruitment of CDK9 to phosphorylate the C-terminal domain of Pol II [191]. However, genetic screenings, looking for cellular transcription factors involved in HIV-1 latency, recently identified BRD4 as an inhibitory factor of LTR expression [192,193]. Indeed, JQ1, a novel and selective small molecule inhibitor of bromodomain and extraterminal (BET) protein family, with highest specificity for BRD4, has been found by several groups as able to induce proviral expression in some cell line models of latency [194,195,196,197]. This is in accordance with the ability of BRD4 to interact with both subunits of P-TEFb and to compete with Tat for binding to P-TEFb at the HIV-1 promoter [198]. Interestingly JQ1, also exhibits anti-inflammatory properties possibly not correlated with BRD4 or other BET protein inhibition. Moreover, similarly to HDACis, it synergizes with prostratin and suppresses T cell proliferation by down-regulating T cell activation genes, including CD3, CD28, and CXCR4, and also up-regulates chromatin modification genes, including SIRT1, HDAC6, and multiple lysine demethylases (KDMs) [194,196]. These effects and the low toxicity would provide a rationale for its use as reactivating agent. However, so far, studies in primary cells and patients gave mixed results. JQ1, indeed, reactivated the virus in one of three patients’ samples in one study [194], while JQ1 alone showed little to no effect on HIV-1 gene expression in the setting of newly infected primary human T cells, even if it increased PMA or prostratin effect [195,196]. The outcome was also variable in primary CD4^+^ T cell models of latency where JQ1 was able to reactivate latent virus in the model developed in the Siliciano lab but was inactive in another primary latency model developed by Planelles’ group [199]. Interestingly, the same authors found that, in addition to BRD4, a second BET protein, BRD2, regulates HIV latency even if with different molecular mechanisms. Further studies to elucidate the differential activity of JQ1 in different cell models of latency and its exact mechanism of action are necessary in order to evaluate whether JQ1 may progress to clinical application.

Recently the synthetic lipopeptide Pam3CSK4, a Toll-like receptor-1/2 agonist, has been shown to reactivate latent HIV-1 in the primary latency model developed by Planelles’ group and in cells isolated from aviremic patients. Interestingly, this reactivation was NF-κB, NFAT and AP-1-mediated and required pTEFb activity [200]. The importance of this work lies on the possibility to engage these three transcription factors together, thus mimicking the synergistic activation of HIV-1 transcription obtained during antigen-mediated stimulation of T cells, but without T cell activation or proliferation. In particular, the possibility to stimulate the activation of the NF-κB transcription factor in cells resembling the T CD4^+^ resting central memory phenotype is crucial for the “shock and kill”strategy since these cells have been thought to be refractory to the activation of NF-κB by stimuli acting positively on activated T cells and in T cell line models of HIV-1 latency.

Novel compounds whose mechanism of action in reverting latency still remains to be identified has been revealed through the use of high-throughput screening of chemical and siRNA libraries in *in vitro* cell model systems of HIV latency. Among these compounds, the screening performed with the latently infected primary CD4^+^ T cell model developed in the Siliciano laboratory identified disulfiram and two classes of quinolines derivatives of quinoline-8-ol, that act as antilatency compounds. Disulfiram, a zinc-chelating agent, FDA-approved for use in humans to treat alcoholism, was recently reported to turn on HIV-1 transcription without global T cell activation or cytokine release in primary cell model of latent HIV [201]. At the molecular level, disulfiram via reduction of PTEN protein indirectly stimulates the Akt pathway leading to the release of P-TEFb [202]. An ongoing single-arm pilot clinical trial (NCT01286259) enrolling 14 patients will try to assess whether short-term administration of disulfiram will impact HIV reservoir in patients under ART. Initial results did not provide clear evidence of an effect, showing a rapid and modest increase in plasma HIV-1 RNA in all participants but a very modest reduction in the size of the latent reservoir [203]. It is thus unclear whether disulfiram can be clinically significant in reducing the viral reservoir or should be used in combination with other reactivating agents.

Using the same model system, the group of Siliciano recently identified two classes of quinolines derivatives of quinoline-8-ol, a bivalent cation chelator, adducts of 5-chloroquinolin-8-ol, and quinolin-8-yl carbamates, that induce HIV-1 expression without cell activation [204]. Although the mechanisms of action of these compounds remains to be determined, the absence of general T cell activation in spite of viral reactivation indicates that they could represent a new class of drugs for treating latent HIV-1 infection.

The new small molecule antiviral 6 (AV6) was similarly identified in a high-throughput screen of 200,000 compounds in the SupT1 lymphoid cell-line model of HIV latency. It requires NF-AT to activate latent HIV-1 in several clonal T cell lines and in infected primary CD4^+^ T cells, albeit not latently-infected ones. Again, the observation that it does not induce general T cell proliferation or activation and also synergizes with VPA [205] is in favor of its future development as an antilatency drug.

While the number of agents active *in vitro* in disrupting latency is increasing, their validation in primary cell models and cells from patients as well as the elucidation of their mechanisms of action have to be assessed before their designation as candidates for employment *in vivo*.

### 4.2. Immune-Based Therapies

Immune-based therapies have been proposed in combination with reactivating compounds to enhance the clearance of the reactivated latently-infected cells as well as to improve host immune responses. After reactivation, is indeed not established whether the reactivated cells are killed by cytopathic effects or recognized and eliminated by the immune system [206]. Moreover, a hallmark of chronic infection and progression to AIDS is the persistent immune activation, even after years of suppressive ART, characterized by a sustained innate response that is believed to contribute more to HV-1 pathogenesis than to protection [106,207,208,209,210].

Among immune-based therapies the use of cytokines and agents that antagonize negative regulators of immune activation have been considered due to their capacity of both reversing virus silencing and restore immune functions. Therapeutic vaccination and anti-inflammatory drugs should, instead, aid in improving the quality and/or magnitude of HIV-1-specific immune responses promoting cytotoxic T cell responses blunting viral rebound after ART interruption and in reverting T cell exhaustion thereby leading to better immune control.

The use of cytokines has been envisioned as a therapeutic strategy in individuals on ART for almost 15 years [211,212] due to their capacity to stimulate HIV-1 replication and interfere with mechanisms responsible for HIV-1 latency [213]. Several cytokines including IL-2, IL-7, and IL-15, that have putative role in HIV-1 control have been, so far, proposed and/or tested in humans. Despite initial optimistic results obtained in a non-randomized study suggesting that intermittent IL-2 plus ART could led to a reduction in the pool of latently-infected cells [212], subsequent larger studies indicated that combined treatment with IL-2 and ART did not actually reduce HIV-1 reservoirs [214,215,216]. The possibility of using IL-2 in combination with the anti-CD3 antibody OKT3 was also tested in a small study (three patients). This regimen however resulted in significant side effects being highly toxic, due to profound T cell activation and proliferation and induction of antibodies against OKT3, without having any apparent positive effect on the latent reservoir [217,218]. Moreover, *in vivo* IL-2 administration to HIV-infected patients also led to peripheral expansion of a population of long-lived CD4^+^CD45RO^+^CD25^+^ cells that express high levels of FoxP3 indicating a regulatory T cells phenotype [219]. These cells by inhibiting CD8^+^ T cell-mediated cytotoxicity could thus compromise the subsequent elimination of reactivated cells by immune means.

IL-7 is another member of the γc cytokine family that has been considered as a promising candidate in HIV eradication strategies. Initially it was shown that IL-7 activated *in vitro* latent provirus from resting CD4^+^ T lymphocytes from HIV-1-infected patients on ART being more effective than either IL-2 alone or IL-2 combined with phytohemagglutinin (PHA) [220] and also to selectively induce distinct proviral quasispecies as compared with IL-2 suggesting a direct effect on the latent reservoir. Subsequent testing of the IL-7 activity in primary cell models of latency however, gave contrasting results. While effective in reactivating virus in an *in vitro* primary model of CCL19-induced HIV-1 latency [221], it has proven ineffective in reducing the reservoir size, despite partial reactivation of latent HIV-1, in another primary model of latency. More importantly, IL-7 has proven to favor homeostatic proliferation of the TTM subset of latent memory T cells [77]. A major bias in the use of IL-7 therapy is also coming from clinical trials aimed at evaluating safety and efficacy of IL-7 administration in HIV-1-infected individuals. Results indicated that this cytokine induces expansion of circulating naive (TN) and central memory (TCM) CD4^+^ and CD8^+^ T cells that could favor enhancement of immune effector function, but also determine a transient increase in viral replication [222,223,224]. As in the case of IL-2, the “blips” of viremia observed during IL-7 administration appear to be transient and consist in a virus similar to the viruses present prior to therapy [225] suggesting that the low level of viremia induced by IL-7 mainly depends on transient induction of the virus from a preexisting pool of productively infected cells rather than activation of silent quasispecies in the reservoir. These results together with the recently reported findings from the NCT00099671 Clinical Trial that, when administered to virally suppressed subjects, IL-7 led to the rapid proliferation of memory CD4^+^ T cells, which resulted in a 70% increase in the absolute number of circulating CD4^+^ T cells harboring integrated HIV DNA four weeks after therapy [226], challenge the use of IL-7 in eradication strategies. Currently, one trial (NCT01019551) the ERAIMMUNE 01 that will test IL-7 in combination with anti-HIV-1 vaccine and ART intensification with raltegravir plus maraviroc, to prevent proliferation or reseeding of the latent reservoir, is ongoing.

IL-15, is another member of the IL-2 family of cytokines, that could be a candidate for immunotherapies aimed at increasing immune-mediated elimination of infected cells during ART and as such it is currently tested in three clinical trials as an immune adjuvant for poorly immunogenic HIV-1 vaccines [227]. Whether IL-15 can act also as reactivating agent is under evaluation. To date, only a recent abstract reports that IL-15 also induces viral reactivation *in vitro* as a consequence of differentiation of memory CD4^+^ T cells with a minimal effect on proliferation [228]. In *in vivo* studies in virally suppressed chronically SIV infected macaques, however, IL-15 delayed viral suppression and failed to enhance ART-induced total and antigen-specific CD4^+^ T-cell reconstitution at mucosal and lymphoid sites. Moreover, upon treatment interruption, IL-15-treated macaques lose CD4^+^ T cells faster than those receiving ART alone [229]. Thus, further studies are required to elucidate the effective potential of IL-15 as a candidate to deplete the HIV reservoir.

As another immune-mediated approach, a recent study found that the use of pegylated interferon alpha-2A (clinical trial NCT005948) controlled HIV replication and decreased HIV-1 integration in patients after interruption of ART [230].

As outlined above, immune dysfunctions stemming from chronic immune activation including progressive functional impairment of T cells, called immune exhaustion, and continuous inflammation are hallmarks of HIV infection even in the presence of ART [207,210,231,232,233] and significantly contributes both in the initial establishment and in the subsequent maintenance of the viral reservoir [209]. Thus, strategies aimed at the recovery of immune system functionality are highly pursued. An attractive immunological target is programmed cell death protein 1 (PD-1), a receptor known for its role in immune exhaustion as reviewed in [234,235,236]. PD-1 receptor is up-regulated in HIV-specific and non-specific CD4^+^ and CD8^+^ T cells limiting their functions [237,238,239,240] and in the compartment of memory T cells those expressing high levels of PD-1 contain more proviral HIV-1 DNA than PD-1 low cells [77]. Likewise, a consistent association between the frequency of PD-1-expressing cells and the size of the reservoir has been found *in vivo* in patients on c-ART [237,241]. Consistently, PD-1 is expressed at lower levels in Elite Controllers and Long-Term Non-Progressors (LTNPs) compared to typical progressors [242]. These findings suggest that PD-1 high cells constitute a preferential reservoir for the virus and that PD-1 inhibition may provide beneficial effects also in reducing the latent reservoir. Thus, PD-1 receptor is currently the focus of several studies aimed at testing whether blocking this negative regulator of immune activation may both activate HIV-1 transcription and reverse immune exhaustion by relieving a functional block on virus-specific CD8 memory T cells [236,243]. To date, only preliminary *in vitro* results have indicated that the interaction between PD-1 and its ligand may suppress HIV-1 thus favoring latency while blocking PD-1 engagement results in increased viral replication and may therefore reactivate latent virus [244].

*In vivo*, anti-PD1 antibodies have been tested in non-human primate models of HIV infection with positive effects on immune system recovery. The results indicate improved immune responses in the blood and in the gut, associated with significant reductions in plasma viral load, greater antibody responses to both SIV and non-SIV antigens and prolonged survival of SIV-infected macaques [245,246]. In addition, PD-1 blockade reduced persistent immune activation with evidence for restoration of mucosal barrier integrity and bacterial translocation [247]. These preclinical results thus provide a rationale for targeting PD-1 in HIV infected patients in combination with anti-retroviral therapy in order to both reduce the reservoir size and increase anti-HIV-1 specific immune responses. In humans, blocking the interaction between PD-1 and its ligand, PD-L1, is currently being evaluated in two large Phase I trials in patients with advanced cancer [248,249]. Results are promising regarding safety but have so far provided variable responses and clinical responses only in a small proportion of patients. Separate trials have to be performed in order to assess how blockade of the PD-1 pathway will impact systemic inflammation present in HIV-1 patients.

A study of an AIDS Clinical Trial Group (ACTG 5301) testing the safety and efficacy of PD-1 blockade to reduce the latent HIV reservoir in patients in c-ART is currently under development.

Lymphoid tissue damage is characterized by collagen deposition and fibrosis, occurring as a consequence of high inflammation prior to the initiation of ART [250]. To reduce lymphoid fibrosis that does not normalize with ART [251], limits immune reconstitution [252] and specific anti-HIV-1 responses, a proposed approach is the use of antifibrotic agents.

Angiotensin-converting enzyme inhibitors and angiotensin receptor blockers, that have shown antifibrotic effects through the inhibition of transforming growth factor-β in different clinical settings [253,254], are currently being tested in phase I and II clinical trials (NCT01535235; ACTG 5317) in the context of HIV infection. Their capacity to improve HIV-specific immune responses and to reduce the viral reservoir in lymphoid tissues is also tested.

Two peroxisome proliferator-activated receptor agonists, plioglitazone and leflunomide, are other promising anti-inflammatory molecules, well tolerated and effective in the treatment of chronic inflammation [255] that might be useful in reducing metabolic syndromes associated with prolonged ART [256]. The therapeutic efficacy of these approaches in decreasing virus production and viral reservoirs size, however, remains to be demonstrated.

Immunotherapies aimed at restoring a cellular immunity that has been demonstrated important to control the HIV-1 reservoir size [206,257,258,259] include therapeutic vaccination. This strategy could re-stimulate CTL responses, mimicking the situation of the minority of patients who control viral replication without treatment and do not progress to AIDS [260,261,262]. This approach could also be useful in reactivating virus as the HIV-1 env and pol antigens were reported to activate most of CD4^+^ T cells harboring proviral DNA and to induce HIV-1 replication [263]. In the NCT00107549 clinical trial patients were immunized with a poxvirus vaccine engineered to express HIV-1 antigens and a significant, albeit transient, decrease in replication-competent HIV-1 in the resting T-cell reservoir was observed [260]. Currently, the ongoing Eramune-02 clinical trial is testing whether a DNA prime plus the polyvalent HIV-Gag, Pol, Nef, and Env vaccine (HIV-rAd5 vaccine), can reduce the viral reservoir size in patients undergoing an ART-intensification regimen.

Based on the observation that several vaccination regimens and pathogen infections have been shown to transiently increase viral RNA in plasma of HIV-1-infected patients in ART, recently, the use of Toll-like receptors (TLRs) agonist has been suggested to both reactivate HIV-1 from latently infected cells and to boost HIV-specific cytotoxic CD8^+^ T cell immunity [200,264]. Interestingly, as reported above, at least the TLR1/2 agonist appears to be selective for latent, integrated viruses and viral reactivation in the absence of T cell activation and proliferation [200]. However, since TLR activation, with release of type I IFNs and expression of IFN-inducible-genes may substantially contribute to systemic immune activation in HIV-1 infection, the use of TLR agonists must be carefully evaluated even in combinatorial approaches. Consistently, the use of TLR antagonists, as chloroquine, has on the contrary, been considered to lower immune activation. Treatments with hydroxychloroquine or chloroquine have been evaluated in the clinical setting in ART-treated [265] and ART-naive patients [266] and reported to reduce immune activation but with little or no effect on CD4^+^ T cell recovery. In a randomized trial in naive patients non-progressors, however, hydroxychloroquine treatment resulted even in the worsening of CD4^+^ T cell loss [267]. A phase II clinical trial (NCT00819390) to test the safety and tolerance of chloroquine in people infected with HIV-1 and to determine whether chloroquine treatment of HIV-1-infected patients is able to reduce HIV-1-induced immune activation has been just completed, but the results are not available yet.

### 4.3. Gene Therapy Approaches

Following the report of the “Berlin patient”, gene therapy strategies to eliminate the virus from infected cells or to create HIV-1-resistant cells have received significant attention as recently reviewed in [38]. Replication of the “Berlin patient” therapy is however a challenging goal in that donors with the CCR5Δ32 mutation are rare and procedures associated with bone marrow transplantation (BMT), as irradiation and chemotherapy, make allogeneic hematopoietic stem cell transplant a feasible approach only in individuals with cancer as the “Berlin patient”.

Even if a recent report [268] indicates significant reduction, and perhaps elimination, of latent HIV-1 reservoirs following BMT from normal, CCR5 wild-type donors, eliminating the necessity of donor selection for the rare CCR5 Δ32 mutation, nevertheless, the risks associated with BMT still make this approach of limited applicability for HIV patients.

Alternative approaches to BMT involve the engineering of a patient’s own cells through the generation of a CCR5 deletion followed by an autologous transplant with these cells that are resistant to infection or direct deletion of the virus from infected cells. Targeting of HIV coreceptors and pro-viral sequences can be achieved through the use of DNA-editing enzymes [269]. These include zinc-finger nucleases (ZNFs) [270], transcription activator-like effector nucleases and homing endonucleses [269,271,272].

ZFNs have been used to manipulate CCR5 expression in CD4^+^ T cells and precursor cells [270,273]. One clinical trial just completed [274] and two ongoing (NCT01044654, and NCT01252641) [275,276] address the safety and efficacy of CCR5-ZFN-treated autologous cells, known as SB-728-T, in patients with chronic aviremic HIV infection under ART, including effects on CD4^+^ T-cell counts, viral load, and the ability of the cells to localize to anatomical reservoirs. The initial results are encouraging in terms of safety, expansion and persistence, in that not only engineered cells persist in the body but also decline significantly less than non-engineered cells after ART interruption. Moreover a decline in HIV-1 DNA level in the blood of most patients was observed suggesting a decrease in the reservoir size. Such an approach may be safer and more easily applicable on a larger scale compared to the use of autologous CCR5-modified hematopoietic stem cells.

However, these cells do not protect against CXCR4-tropic viruses and may drive selection for either X4-specific or dual-tropic HIV-1 viruses [277,278]. ZFNs targeting CXCR4 in CCR5∆32 CD4^+^ T cells has been recently described and preclinical studies in cell lines and humanized mice indicate protection to both R5- and X4-tropic HIV [279,280]. These data thus provide a rationale for simultaneous disruption of the HIV-1 coreceptors, as a useful approach for the long-term, drug free treatment of established HIV-1 infections. The deletion of CXCR4 on immune function is, however, still poorly understood, and the effects of simultaneous loss of both receptors remain to be explored. Moreover, several other drawbacks may be connected with this technology, as recently reviewed [269].

Anti-HIV-1 ribozymes or antisense RNA oligonucleotides are other gene-based approaches in phase I and phase II clinical trials, that have been shown to be safe and to reduce viral load [281]. Preclinical studies on a therapy known as Triple-R, which combines expression of an anti-CCR5 ribozyme, anti-Tat and Rev short hairpin RNAs, and a transactivation response element decoy RNA containing a nucleolar localization tag, have indicated an effect at protecting cells from HIV-1 infection in *in vitro* and mouse model, but in the phase I clinical trial NCT01153646, this approach has been demonstrated negligible on HIV infection [282].

Thus, although these therapies appear in general to be safe, their efficacy *in vivo* in humans has yet to be proven.

## 5. Conclusions and Challenges

In spite of considerable achievements in the control of HIV-1 infection through ART, eradication of HIV-1 still represents a major challenge for the scientific community, with many barriers still remaining in finding a cure for AIDS.

The major and so far insuperable obstacle, is the presence of small pools of cells harbouring an integrated and replication-competent virus that, as soon as therapy is interrupted, refuel systemic infection and immune cell dysfunctions caused by persistent immune activation that current therapies do not cure. 

The past few years have seen the development of several new therapeutic approaches to successfully control (functional cure) or eliminate (sterilizing cure) the viral reservoirs, but overall eradication protocols, so far tested through preclinical and clinical studies, have yielded unsatisfactory results.

These strategies include viral reactivation followed by the elimination of infected cells through viral cyto-pathogenicity, host immune responses or cytotoxic drugs, as well as improving host immune responses to target and clear residual viremia, engineering patient’s own cells to perform an autologous transplant with cells that are resistant to infection and deletion of the virus from infected cells.

Several agents inducing activation of the silent virus are currently being tested each targeting a different mechanism involved in the establishment/maintenance of latency. These include HDAC-, DMT- and HMT-inhibitors, PKC and P-TEFb activators. As partial and/or disappointing results in reducing the reservoir have been obtained with single agents, effective activation of the entire latent pool, including reservoirs in privileged anatomical sites, may ultimately require a combination of drugs or unique therapeutic approaches. Nevertheless major obstacles in the clinical use of even single compounds must be overcome before considering their combination in humans. These concerns include relative toxicity at therapeutically effective doses. This is the case of compounds such as PKC activators, that at concentrations required for efficient HIV-1 reactivation, may be associated with uncontrolled T cell activation with a great risk of inducing a cytokine storm, with detrimental consequences for the patient. New compounds that more selectively target specific subtypes of PKCs or act on steps downstream in the signaling pathways might reduce this risk.

Similarly, the use of compounds that affect cell transcriptional machinery as epigenetic modulators, in addition to toxicity, poses also the possibility of stirring endogenous retroelements that are kept at bay by the same mechanisms that hide HIV-1 [283,284]. It has been also reported that recombinant HIV-1 Tat protein activates the expression of an endogenous retrovirus from the HERV family, in lymphocytes and monocytic cells [285] strictly linking HIV-1 reactivation to endogenous retroviruses expression. Since it is estimated that 7%–8% of the human genome is derived from retroviral and retrotransposon sequences lying dormant, loss of control of these elements may direct aberrant gene expression contributing to a wide range of human diseases [286].

Reactivation of viral expression is, on the other hand, not sufficient to eliminate the reservoir since it is very likely that reactivated cells are not eliminated by cytopathic effects or host cytolytic mechanisms [206]. Thus, combinatorial approaches that can effectively induce reactivation of the latent reservoirs and enhance specific immune responses to control virus replication and eliminate virus-infected cells should be considered. Similarly to epigenetic approaches that have been borrowed from cancer therapy, combinatorial approaches already used in cancer therapy may provide an interesting approach [287].

Genetic strategies aimed at the elimination of cells expressing HIV proteins or at creating HIV-1-resistant cells and immune-based therapies to reverse immune exhaustion and to stimulate HIV-1-specific cellular immunity are also all currently under active clinical investigation and should be considered in combinational anti-latency therapies.

Despite the successes obtained, so far, in finding anti-latency agents, many critical and unsolved issues in their clinical use, however, remain. Though not discussed here, but recently reviewed elsewhere [288], a major challenge in the evaluation and testing of new therapeutics is represented by their validation in suitable models of latency that may recapitulate latency *in vivo*. Both cell lines and the several primary cell models of latency so far developed, do not completely mirror results obtained from patient cells and experimental results often vary from one model to the other. This is probably due to the fact that they model single aspects of latency. Thus, there is an urgent need for developing cell and non-human primate animal model systems that faithfully represent the *in vivo* situation of latently-infected cells in the setting of ART, to provide a fuller and standard representation of persistent HIV infection and allow rigorous preclinical testing of any new pharmacologic approach. Similarly, universally employed and standardized methods for measuring latent HIV infection to monitor the impact of eradication strategies in clinical trials are still lacking. Latently-infected cells are very rare *in vivo* (one in a million) and thus hard to study, and current assays that measure the size of the reservoir require a large amount of patient blood and are imprecise and not useful for therapeutic monitoring. Much effort is ongoing to find new reliable methods that are economic, not time-consuming and that can measure replication-competent virus, to check real effectiveness of treatments under investigation with reproducibility in different laboratories.

Last, but not least, ethical issues need to be addressed. Considering the relatively normal life of patients in ART, new clinical approaches that reactivate the virus in individuals already successfully treated must provide minor and limited side effects during the treatment.

Despite all these drawbacks, the recent studies showing that very early treatment with ART [28,29] can reduce the size and complexity of the latent reservoir possibly leading to post-treatment control of the infection, together with data obtained in a chronically SIVmac251-infected macaques showing a drug-free post-therapy control of the infection even in the chronic phase of the disease [36], the never ending campaigns of prevention and progresses made in the last years also in gene therapy approaches, constitute the basis for evaluating safer and more effective combination approaches to HIV-1 remission and hopefully cure.
